# Is Intestinal Dysbiosis-Associated With Immunosuppressive Therapy a Key Factor in the Pathophysiology of Post-Transplant Diabetes Mellitus?

**DOI:** 10.3389/fendo.2022.898878

**Published:** 2022-07-07

**Authors:** Quentin Faucher, Manon Jardou, Clarisse Brossier, Nicolas Picard, Pierre Marquet, Roland Lawson

**Affiliations:** ^1^ University of Limoges, Inserm U1248, Pharmacology & Transplantation, Limoges, France; ^2^ Department of pharmacology, toxicology and pharmacovigilance, Centre Hospitalier Universitaire (CHU) Limoges, Limoges, France

**Keywords:** post-transplant diabetes mellitus, type 2 diabetes mellitus, immunosuppressant, intestinal dysbiosis, bacterial metabolites

## Abstract

Post-transplant diabetes mellitus (PTDM) is one of the most common and deleterious comorbidities after solid organ transplantation (SOT). Its incidence varies depending on the organs transplanted and can affect up to 40% of patients. Current research indicates that PTDM shares several common features with type 2 diabetes mellitus (T2DM) in non-transplant populations. However, the pathophysiology of PTDM is still poorly characterized. Therefore, ways should be sought to improve its diagnosis and therapeutic management. A clear correlation has been made between PTDM and the use of immunosuppressants. Moreover, immunosuppressants are known to induce gut microbiota alterations, also called intestinal dysbiosis. Whereas the role of intestinal dysbiosis in the development of T2DM has been well documented, little is known about its impacts on PTDM. Functional alterations associated with intestinal dysbiosis, especially defects in pathways generating physiologically active bacterial metabolites (e.g., short-chain fatty acids, trimethylamine N-oxide, indole and kynurenine) are known to favour several metabolic disorders. This publication aims at discussing the potential role of intestinal dysbiosis and dysregulation of bacterial metabolites associated with immunosuppressive therapy in the occurrence of PTDM.

**Graphical Abstract:**
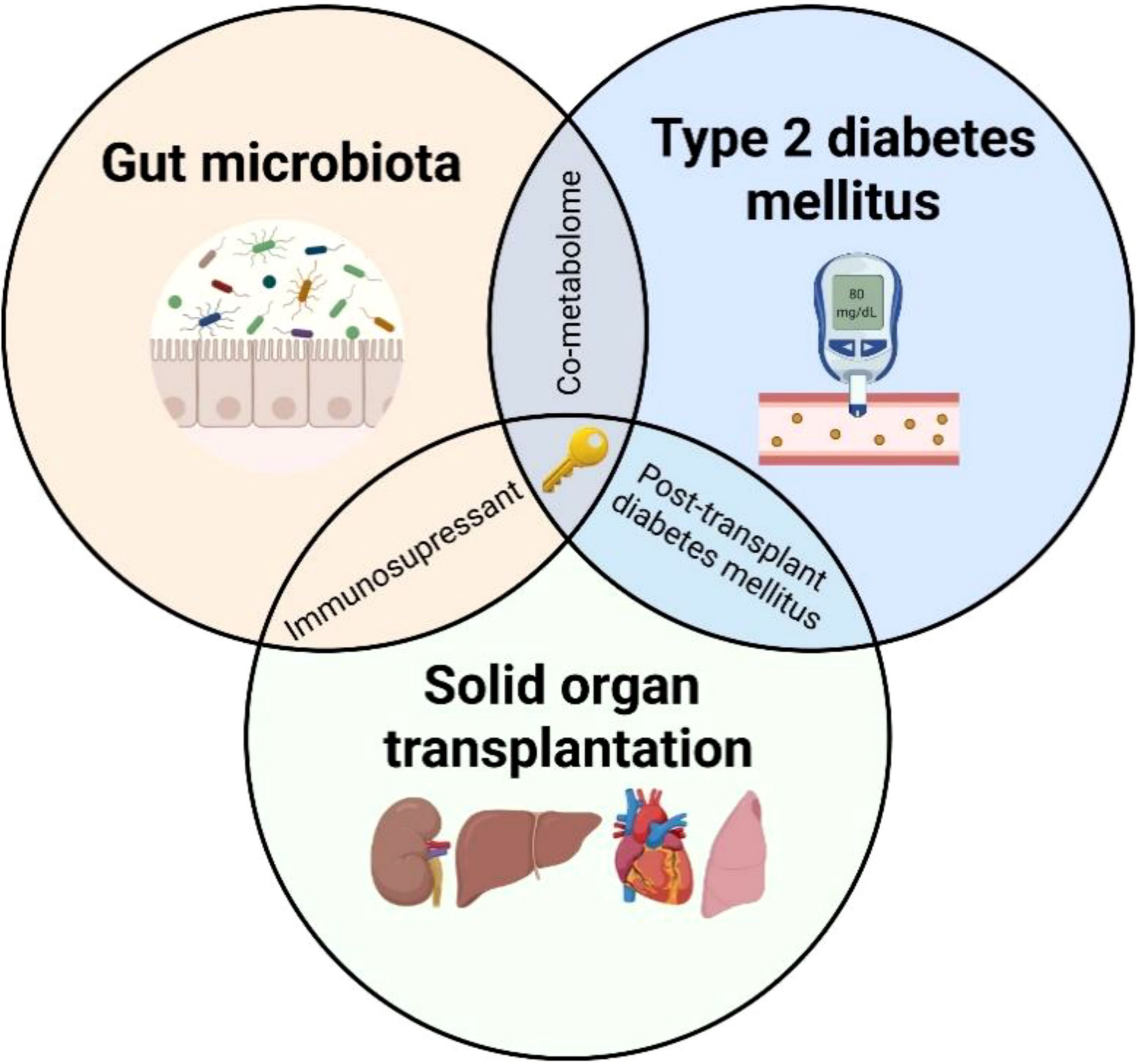


## 1 Introduction

Solid organ transplantation (SOT) is the best replacement therapy in numerous cases of organ failure or end-stage organ dysfunction (*e.g.*, kidney, liver, heart, or lung). Immune tolerance of the transplanted organ requires a complex and life-long immunosuppressive therapy, involving combinations of drugs from six main classes: 1) anti-proliferative agents (azathioprine, mycophenolic acid); 2) calcineurin inhibitors (cyclosporine, tacrolimus); 3) mammalian target of rapamycin (mTOR) inhibitors (sirolimus, everolimus); 4) co-stimulation blockers targeting CD80/CD86 (belatacept); 5) anti-lymphocyte polyclonal or monoclonal antibodies (*e.g.*, anti-thymocyte globulins, basiliximab); and 6) corticosteroids (*e.g.*, prednisolone) (1, 2). The immunosuppressive strategy along time consists of: an induction phase that involves anti-lymphocyte antibodies, corticosteroids and the use of higher doses of “maintenance” immunosuppressants such as antimetabolites and calcineurin inhibitors; a life-long maintenance phase with different combinations of classes 1 to 4 with or without corticosteroids (3); and treatment of rejection, using boluses of corticosteroids, anti-thymocyte globulins, increased doses of maintenance drugs, and potentially other drugs in case of antibody-mediated rejection (ABMR) (4). Unfortunately, these therapeutic regimens increase the risk of opportunistic bacterial, viral, and fungal infections (5) and expose patients to numerous adverse effects and several metabolic disorders.

Post-transplant diabetes mellitus (PTDM) is a common and deleterious co-morbidity, which significantly contributes to adverse outcome. PTDM is an endocrine and metabolic disease characterized by a dysfunction of pancreatic β-cell, insulin resistance, and high blood glucose. Among the risk factors of PTDM, several are common with type 2 diabetes mellitus (T2DM) (*e.g.*, age, abdominal obesity) whereas others are transplant-specific (*e.g.*, immunosuppressive drugs with diabetogenic properties, infection, and post-transplant weight gain) (6, 7). Immunosuppressive drugs can influence gut homeostasis through an impact on intestinal epithelial cells or organs associated with the digestive tract and induce changes in the richness and diversity of the gut microbiota. This drug-microbiota relationship may directly or indirectly affect the anti-rejection treatment efficacy as well as disrupt the microbiota balance and favour the development of metabolic disorders (8).

Although preventative and therapeutic strategies are being deployed to prevent PTDM, its incidence remains high. To improve the effectiveness of such strategies, it is necessary to better understand PTDM pathophysiology. Our hypothesis is that modifications of the gut microbiome, also named intestinal dysbiosis, a well-known contributor to type 2 diabetes mellitus (T2DM) in the non-transplant population, play an even larger role in the pathogenesis of PTDM. To substantiate this hypothesis, we herein provide a picture of the impact of SOT and immunosuppressive therapy on the gut homeostasis including gut microbiota. Subsequently, we discuss the potential role of intestinal dysbiosis in the development of PTDM based on knowledge gained from T2DM and provide arguments in favour of monitoring the microbiota diversity and function to decipher PTDM pathophysiology.

## 2 Current Knowledge on Post-Transplant Diabetes Mellitus

### 2.1 Diagnosis and Incidence

PTDM is a one of the most important comorbidities associated with SOT. The evaluation of its incidence among transplant patients has suffered from the lack of a consensual definition. The first international consensus guidelines about new-onset diabetes after transplantation (NODAT) were published in 2003 (9). A second international consensus conference was held in 2013 to review the criteria and available evidence and proposed an update to the previous guidance (10). Among many recommendations, the first was to enlarge the notion of NODAT to that of PTDM. PTDM encompasses several complex clinical entities and includes hyperglycemia in the post-transplant period resulting from known or unknown pre-existing diabetes, insulin resistance or insulinopenia, transient hyperglycemia, and NODAT. Therefore, this definition encompasses pre-transplant in addition to “new-onset” diabetes (10). Currently, the different diagnostic criteria for PTDM (**Table S1**) are based on those of the American Diabetes Association and on the World Health Organization criteria for non-transplant patients (11).

The incidence of PTDM ranges from 10 to 40% depending on the transplanted organ. Recently, PTDM has been reported to occur in 10-20% of kidney, 20-40% of liver or lung, and 20-30% of heart transplant recipients (6). These large ranges may be explained by the type of organ, the presence of modifiable and non-modifiable risk factors, and the follow-up duration (5). The development of diabetes in transplant recipients is associated with a higher risk of graft failure, patient death, and other adverse outcomes (*e.g.*, cardiovascular disease and infection) (12). More specifically, PTDM is associated with a higher incidence of cardiovascular disease for liver and kidney transplant recipients (13, 14). Heart transplant recipients with PTDM present an increased risk of comorbidities and premature death (15). Moreover, PTDM in lung transplant recipients is associated with shorter survival (16).

### 2.2 Pathogenesis and Risk Factors

PTDM shares common features with type 2 diabetes mellitus (T2DM) such as insulin resistance, hypertriglyceridaemia, cardiovascular events, and chronic low-grade inflammation. Hyperglycaemia in PTDM is associated with pancreatic β-cells dysfunction and decreased insulin sensitivity (6, 17). Studies in PTDM patients reported impaired insulin-mediated glucose uptake in peripheral tissue, impaired insulin-mediated suppression of hepatic glucose output (18) and insufficient incretin release leading to an increase of glucagon release by the pancreas (19). Pre-existing risk factors common to PTDM and T2DM, such as age, abdominal obesity, family history, and ethnicity favour the development of PTDM. The morphotype in the pre-transplant period could predict to some extent the development of PTDM in kidney transplant recipients (20). Moreover, several T2DM-associated single nucleotide polymorphisms (SNPs) in interleukin genes (*e.g.*, IL-7R, IL-2, and IL-17R) are associated with increased pro-inflammatory pathways and PTDM development (21, 22).

Among transplant-related risks factors, numerous studies have demonstrated the involvement of certain immunosuppressive drugs in the development of NODAT. Calcineurin inhibitors dysregulate the function and growth of pancreatic β-cells through the calcineurin/NFAT signalling pathway. Corticosteroids are known to decrease the secretion of, and sensitivity to, insulin (23). Sirolimus favours insulin resistance and decreases pancreatic β-cell proliferation too (24). Consistently, mTOR inhibitors are associated with a higher risk of PTDM (25). Above all, recent analysis pointed toward the contribution of immunosuppressants to the dysregulation of genes involved in insulin production and secretion (24). Viral infections are a source of inflammation and represent yet another risk factor of PTDM. Numerous studies have reported an increased risk of PTDM in kidney and liver transplant recipients positive for the hepatitis C virus (HCV) (26–28) or in kidney transplant recipients positive for the cytomegalovirus (CMV) (29). Although associations between these viral infections and PTDM are generally attributed to the promotion of a pro-inflammatory environment as well as to pancreatic β-cell dysfunctions, extensive studies are missing.

Actually, the preventative strategies against PTDM involve lifestyle (e.g., dietary, physical activity) modifications or adapted immunosuppressive regimens (30). However, the frequency of PTDM has not decreased significantly over the last decade, suggesting that current knowledge is not sufficient and that uncharacterized phenomena contribute to PTDM. Several risk factors presented above (*e.g.*, obesity, immunosuppression, infection) are accompanied by an imbalance in the diversity of the gut microbiome, called intestinal dysbiosis, metabolic disorders and increased intestinal permeability. These alterations are well known to favour T2DM in the non-transplant population (31). Therefore, the drastic dynamic changes of the gut microbiota during SOT may contribute even more to the pathogenesis of PTDM.

## 3 Alterations of Gut Homeostasis in Solid Organ Transplantation

**Figure 1:**
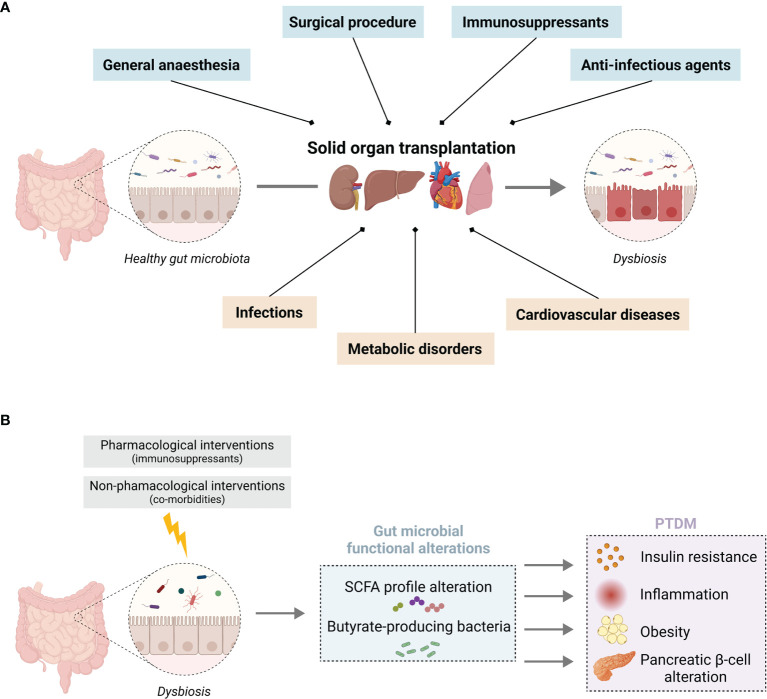
Solid organ transplantation associated pharmacological and non-pharmacological interventions on gut microbiota homeostasis **(A)** Overview of the impact of SOT on gut microbiota homeostasis associated with (blue) the surgical procedure, anti-infectious prophylaxis, immunosuppressants, and (orange) SOT-related co-morbidities. **(B)** Putative consequences of an imbalance in SCFA-producing bacteria induced by SOT, which favour the development of PTDM. Illustration created with BioRender.com.

Gut homeostasis is highly dependent on the intimate crosstalk between the gastrointestinal tract and the gut microbiota. The gut microbiota represents the populations of commensal microorganisms that reside in the gastrointestinal tract and participate in the intestinal barrier integrity. Recent scientific advances have underlined the fundamental role of this microbiota in the regulation of the immune system, as well as the close relationship between intestinal dysbiosis and the occurrence of numerous local or systemic diseases (mainly cardiovascular or metabolic disorders) (32, 33). The gut microbiota may therefore represent an actionable target to improve immune tolerance and long-term graft survival. The diversity, richness, and activity of its resident microorganisms are constantly being modified under the influence of various factors (*e.g.*, genetic, dietary, environmental, and therapeutic). It is worth mentioning that in transplantation, the nature of the transplanted organ, the various pre- and post-transplant pathologies, and multiple therapies accentuate the gut microbiota variability. The dynamic changes of the gut microbiota have been widely studied in some diseases, but very seldom in transplant patients. We summarize below current knowledge about the relationships between the gut microbiota and SOT outcomes (**Figure 1A**), including the impact of the immunosuppressive protocol and the occurrence of post-transplant co-morbidities.

### 3.1 Dynamic Changes of the Gut Microbiota in Transplant Patients

The surgical procedure of transplantation which is generally an abdominal act, represents a high risk of intestinal dysbiosis (34, 35). Regarding the post-transplantation period, a cohort study in kidney transplant recipients reported changes in gut microbial diversity in favour of an increase in Proteobacteria, a phylum that includes potentially virulent pathogens (*e.g., Escherichia coli*, *Klebsiella pneumoniae*, *Pseudomonas aeruginosa*) (36). The faecal microbial diversity was decreased in some patients with post-transplant complications (diarrhoea, acute rejection, urinary tract infection) (37). In addition, the diversity of the gut microbiota was significantly lower and the levels of Proteobacteria higher with abundant *Escherichia coli* in kidney transplant recipients compared to healthy control (38). In liver transplant recipients, this diversity transiently decreased two weeks after transplantation and then gradually increased back to reach the pre-transplantation levels after 5 weeks (39). Anti-infectious agents used in transplanted patients to prevent opportunistic infections, mainly antibiotics, are known to affect the gut microbiota homeostasis and to promote intestinal dysbiosis (36, 40).

### 3.2 Effect of the Immunosuppressive Therapy on the Gut Homeostasis

#### 3.2.1 Interactions Between the Immunosuppressive Drugs and the Gut Microbiota

A recent review from Gabarre *et al.* has provided a thorough overview of the bidirectional interaction between the immunosuppressants and the gut microbiota (8). The use of the anti-proliferative agent, mycophenolic acid, initially known for its antibacterial, antifungal and antiviral properties, is associated to a decrease in the diversity of the gut microbiota in kidney transplant recipients (38, 41). Further investigations in preclinical models have revealed an alteration of the intestinal microbiota in mycophenolic acid-treated mice with an expansion of bacteria belonging to the Proteobacteria phylum (42, 43). In addition, a decrease of bacterial metabolites was also observed in a mouse model of mycophenolate-induced enteropathy (44). Mycophenolic acid is thought to selectively promote the expansion of β-glucuronidase-expressing bacteria of Enterobacteriaceae family (42, 43). The bacterial β-glucuronidase activity promotes enterohepatic recirculation of mycophenolic acid and increases its exposure to intestinal epithelial cells that could probably explain the occurrence of gastrointestinal adverse effects. Regarding corticosteroids, rats treated orally with dexamethasone sodium phosphate showed a decrease in the richness and diversity of their gut microbiota (45). Prednisolone-treated mice showed a reduction in the population of Bacteroidetes and an increase in Firmicutes in faecal samples (46). For calcineurin inhibitors, a study reported altered microbiota in high-dose tacrolimus-treated mice (8, 47). Another study based on intraperitoneal injection of tacrolimus in rats showed that the relative abundance of several bacterial species in the faeces was decreased (48). The composition of the gut microbiota can affect the metabolism of tacrolimus as some commensal gut bacteria (*e.g., Faecalibacterium prausnitzii*) have been shown to convert it to less potent metabolites (49).

These studies showed in the one hand, that several immunosuppressive drugs induced intestinal dysbiosis leading to change in microbial diversity favouring the increase of opportunistic pathobionts and in other hand, that the gut microbiota influenced the immunosuppressive drugs metabolism and efficacy. However, the characteristics of gut microbiota changes differ across drugs and studies and systematic and longitudinal investigations that could provide insight into these trends are still lacking. However, the above-mentioned review lists the immunosuppressants inducing intestinal dysbiosis and provides an overview of the related changes in the microbiota (8).

#### 3.2.2 Immunosuppressive Drugs Impacts on Intestinal Barrier Integrity and Pancreas Homeostasis

In transplant patients, immunosuppressive drugs can alter the intestinal barrier integrity and favour intestinal permeability. Intestinal permeability is characterized by a loss of the gut epithelial wall integrity allowing different sizes of compounds to enter the systemic circulation (food antigens, commensal or pathogens bacteria, and their metabolites) (50). By using an intestinal epithelial cell line, Qasim *et al.* have demonstrated the potential of mycophenolic acid to alter tight junction proteins expression and distribution and induce intestinal permeability that may be responsible for gastrointestinal adverse effects observed in transplant patients (51). This intestinal permeability could also have deleterious consequences such as chronic systemic inflammation (52). Another study has demonstrated that tacrolimus and sirolimus by inhibiting cell viability and inducing reactive oxygen species formation, can promote major changes in intestinal barrier function (53).

The immunosuppressive drugs can also alter the homeostasis of organs associated with the digestive tract. We herein only focus on the influence of the pancreas homeostasis as it plays a key role in the regulation of nutrient digestion by releasing digestive enzymes and glucose homeostasis (54). Some rare cases of drug-induced pancreatitis have been reported under tacrolimus (55) or mycophenolic acid (56) treatments. However, there is no strong evidence of the direct impact of the immunosuppressive drug on the pancreas homeostasis that could rationalize the occurrence of PTDM. Therefore, the whole impact of the environment (*i.e.*, dysbiosis, immunosuppressive therapy, co-morbidities) could account for the development of PTDM.

### 3.3 Impact of SOT-Related Co-Morbidities on the Gut Microbiota

In the first months post-transplantation, patients are at high risk of developing infections due to a weakened immune system. Serious infections can be caused by commensal or nosocomial bacterial (*e.g.*, *Pseudomonas*, *Klebsiella*, *Escherichia*), viral (*e.g.*, cytomegalovirus, Epstein-Barr virus, influenza) or fungal (*e.g.*, *Candida* or *Aspergillus* species) pathogens (57). *Clostridium difficile*, a frequent perpetrator of nosocomial infection (7.4% prevalence in SOT patients), is linked with the emergence of intestinal dysbiosis (58, 59). Gut microbiome alteration is frequently associated with these infections and is characterized by an enrichment of opportunistic pathogens and a depletion of beneficial commensals (60, 61). For example, a preclinical study has reported variations in gut microbiota diversity in cytomegalovirus-infected mice (62).

The main metabolic complications after SOT include PTDM, non-alcoholic fatty liver disease, dyslipidaemia, and obesity. These metabolic disorders may increase the risk of cardiovascular events (hypertension, coronary artery disease, stroke, arteritis) and affect post-transplant graft outcomes (63–66). For instance, non-alcoholic fatty liver disease is associated with an increase in Proteobacteria, leading to gut dysbiosis (67, 68). Immunosuppressive drugs such as corticosteroids and calcineurin inhibitors can favour hypertension and weight gain (69). This weight gain of SOT patients is critical in post-transplant period, since obesity has been significantly associated with a higher overall mortality and reduced allograft survival particularly in renal transplant patients (70). These metabolic complications can have a deleterious effect on gut microbiota homeostasis. Obesity affect the diversity of intestinal microbiota, with an increase in Firmicutes and a reduction of Bacteroidetes in a mice model (71). Intestinal dysbiosis has been observed in obese people, with an increased Firmicutes-to-Bacteroidetes ratio (72).

In summary, SOT therapy is accompanied by intestinal dysbiosis arising from a combination of factors including lifestyle and dietary changes, surgical procedure, and pharmacological treatments (*e.g.*, anti-infectious prophylaxis, immunosuppressant). Regarding the gut microbiota-diabetes relationship, several studies have demonstrated a huge diversity imbalance in diabetes patients (31). Given the predominant role of this dysbiosis in the pathogenicity of T2DM, the hypothesis of its involvement in PTDM seems strong.

## 4 Potential Involvement of Intestinal Dysbiosis to the Pathophysiology of PTDM

In this section, we will put an accent on the gut microbiome changes observed in transplant recipients that are common with non-transplant T2DM. In this context, we will describe the putative impact of intestinal dysbiosis on the bacterial metabolites and more precisely on short-chain fatty acids (SCFA) and their possible role in the development of PTDM (**Figure 1B**).

As previously mentioned, several immunosuppressive drugs induce intestinal dysbiosis, generally characterised by a reduction in the phylum of Bacteroidetes, contrasting with an expansion of the phylum of Firmicutes (73). The same tendency has been observed in T2DM patients (74). This increased Firmicutes-to-Bacteroidetes ratio was associated with an impairment of nutrient absorption and glucose tolerance, which pave the way for T2DM (73). Moreover, the relative abundance of Proteobacteria is increased in kidney and liver transplant recipients (36, 38), similarly to T2DM patients (74, 75). Some bacterial strains belonging to this phylum are known to favour pathogenic infections (*e.g.*, *Escherichia coli*, *Klebsiella pneumoniae*, *Pseudomonas aeruginosa*) (36, 38). Gut microbiome changes can induce global metabolic disorders. Indeed, the gut bacterial ecosystem ensures the production of microbial metabolites (*e.g.*, SCFA, trimethylamine N-oxide, indole and kynurenine). These metabolites constitute the communication system of the host-microbiome crosstalk (76). Among them, SCFA are the most commonly studied small metabolites produced by the gut microbiota and they represent a robust link between the microbiota and systemic inflammatory diseases, as demonstrated by recent studies (33, 75).

SCFA, and more precisely acetate, propionate, and butyrate, come from the fermentation of indigestible carbohydrates. SCFA are pharmacologically active and can exert their numeral pharmacological functions by either stimulating G-protein-coupled receptors (GPCR41/43/109A) or can be absorbed by colonocytes through multiple monocarboxylate transporters (*e.g.*, sodium-coupled monocarboxylate transporters (SMCT1), monocarboxylate transporter (MCT1/4/5) (77). In the systemic circulation, they can participate in the regulation of several organs (*e.g.*, liver, lung, brain) (33, 78, 79). For example, they can decrease allergic inflammation in the lungs, or can be used as a source of energy by the kidneys, the myocardium and other muscles (80). SCFA facilitate IL-10 synthesis through the polarization of T-cells towards regulatory T-cells, which exhibits anti-inflammatory properties (81). They also exert a positive effect on intestinal cell homeostasis through the maintenance of the epithelial barrier function through the expression of tight junctions that decrease intestinal permeability (82).

Alteration of SCFA profiles has been observed in T2DM patients, with a significant reduction of faecal propionate and butyrate concentrations as compared to control subjects (83). Moreover, a metagenome-wide association study showed a decrease in the abundance of some universal butyrate-producing bacteria in T2DM patients such as those observed in transplant recipients (8, 47, 84). A European cohort study reported the decrease of butyrate-producing bacteria (such as *Roseburia* species and *Faecalibacterium prauznitzii*) in the gut microbiota of women with T2DM (85). These studies provide evidence that T2DM and SOT have in common SCFA-producing taxa alterations leading to decreased SCFA production. Butyrate and propionate influence glucose metabolism through the activation of intestinal gluconeogenesis, while acetate and propionate are substrates for gluconeogenesis and lipogenesis in the liver (86). SCFA play a role in blood glucose concentration by favouring the secretion of incretin hormones, as demonstrated by the butyrate-induced secretion of glucagon-like peptide 1 (GLP1) in a pre-clinical model (87). At the cellular level, the binding of SCFA to GPR41 and GPR43 in the enteroendocrine L-cells leads to increased GLP1 and peptide YY levels, which improve cell sensitivity to insulin and promote satiety. Furthermore, SCFA play a protective role against obesity and insulin resistance (73, 88) and have anti-inflammatory properties, especially butyrate. A decrease of butyrate-producing bacteria may favour metabolic inflammation, which in turn clearly induces insulin resistance and foster T2DM development (75). At the opposite, incubation of neutrophils with SCFA *in vitro* suppressed pro-inflammatory makers increased in T2DM, such as IL-6 and TNF-α (89). A recent experimental study showed that butyrate and acetate protected pancreatic β-cells against stressful conditions and alleviated metabolic stressor-induced apoptosis, mitochondrial dysfunction and ROS overproduction (88). Moreover, by stimulating their receptors, SCFA have been involved in the regulation of pancreatic β-cells function and insulin secretion (90). Overall, these studies demonstrated the important role of SCFA in the pathophysiology of diabetes through various mechanisms of action, which have been well detailed in a recent review (89). The decreased richness of SCFA-producing bacteria in SOT may therefore promote and/or contribute to the development of PTDM.

## 5 Conclusion

This article provides hints in favour of a possible association between intestinal dysbiosis and PTDM, based on complementary and coherent scientific evidence. Further investigations are required to reinforce the descriptive data available for SOT. Characterising gut microbiota composition would help to understand the mechanisms and/or to identify predictive biomarkers of PTDM. The measurement of SCFA concentrations in blood and/or faeces as indicators of the gut microbiota functionality in the pre- and post-transplant periods could also make the case stronger. Moreover, dietary supplementation with SCFA as a postbiotic could restore the gut microbiota homeostasis and constitute a complementary therapy for glucose lowering in PTDM. A recent paper stressed that the implementation of an effective PTDM prevention strategy requires relevant identification of at-risk patients, solid knowledge of its pathogenesis and early detection tools (30). Monitoring the gut microbiota in SOT would comply with these objectives since it could help decipher the pathophysiology PTDM and detect patients at increased risk early.

## Data Availability Statement

The original contributions presented in the study are included in the article/**Supplementary Material**. Further inquiries can be directed to the corresponding author.

## Author Contributions

QF, MJ and CB drafted the paper with the input of all authors. This work has been done under the supervision of NP, PM and RL. All authors contributed to the article and approved the submitted version.

## Conflict of Interest

The authors declare that the research was conducted in the absence of any commercial or financial relationships that could be construed as a potential conflict of interest.

## Publisher’s Note

All claims expressed in this article are solely those of the authors and do not necessarily represent those of their affiliated organizations, or those of the publisher, the editors and the reviewers. Any product that may be evaluated in this article, or claim that may be made by its manufacturer, is not guaranteed or endorsed by the publisher.
