# Supplementary material for: Is Intestinal Dysbiosis-Associated With Immunosuppressive Therapy a Key Factor in the Pathophysiology of Post-Transplant Diabetes Mellitus?
Source: Front Endocrinol (Lausanne). 2022 Jul 7;13:898878. doi: 10.3389/fendo.2022.898878 (PMC9302877; doi:10.3389/fendo.2022.898878)
Supplement: Supplementary file 1 [file Table_1.docx]

**Table S1.** Diagnosis of PTDM

| Fasting plasma glucose ≥ 126 mg/dL (7 mmol/L) |
| --- |
| Random glucose ≥ 200 mg/dL (11.1 mmol/L) with symptoms (polyuria, polydipsia, weight loss, tiredness) |
| Two-hour glucose during an oral glucose tolerance test (OGTT) ≥ 200 mg/dL (≥ 11.1 mmol/L) |
| Hemoglobin A1c (HbA1c) ≥ 6.5% (≥ 48 mmol/mol)* |

*During the first-year post-transplantation, HbA1c is underestimated and requires complementary diagnostics.
